# The View from the Trees: Nocturnal Bull Ants, *Myrmecia midas*, Use the Surrounding Panorama While Descending from Trees

**DOI:** 10.3389/fpsyg.2018.00016

**Published:** 2018-01-25

**Authors:** Cody A. Freas, Antione Wystrach, Ajay Narendra, Ken Cheng

**Affiliations:** ^1^Department of Biological Sciences, Macquarie University, Sydney, NSW, Australia; ^2^Research Centre on Animal Cognition, Centre for Integrative Biology, CNRS, University of Toulouse, Toulouse, France

**Keywords:** navigation, ants, nocturnal, landmarks, foraging, scanning

## Abstract

Solitary foraging ants commonly use visual cues from their environment for navigation. Foragers are known to store visual scenes from the surrounding panorama for later guidance to known resources and to return successfully back to the nest. Several ant species travel not only on the ground, but also climb trees to locate resources. The navigational information that guides animals back home during their descent, while their body is perpendicular to the ground, is largely unknown. Here, we investigate in a nocturnal ant, *Myrmecia midas*, whether foragers travelling down a tree use visual information to return home. These ants establish nests at the base of a tree on which they forage and in addition, they also forage on nearby trees. We collected foragers and placed them on the trunk of the nest tree or a foraging tree in multiple compass directions. Regardless of the displacement location, upon release ants immediately moved to the side of the trunk facing the nest during their descent. When ants were released on non-foraging trees near the nest, displaced foragers again travelled around the tree to the side facing the nest. All the displaced foragers reached the correct side of the tree well before reaching the ground. However, when the terrestrial cues around the tree were blocked, foragers were unable to orient correctly, suggesting that the surrounding panorama is critical to successful orientation on the tree. Through analysis of panoramic pictures, we show that views acquired at the base of the foraging tree nest can provide reliable nest-ward orientation up to 1.75 m above the ground. We discuss, how animals descending from trees compare their current scene to a memorised scene and report on the similarities in visually guided behaviour while navigating on the ground and descending from trees.

## Introduction

Solitary ant foragers moving on the ground are adept at navigating through their environment, both while searching for resources and when returning to their nest. Ants that forage alone show the ability to utilise multiple visual navigational systems to reach desired locations. These mechanisms include path integration using the celestial compass (Collett and Collett, 2000; Wehner and Srinivasan, 2003), systematic search (Wehner and Srinivasan, 1981; Müller and Wehner, 1994; Schultheiss et al., 2013) and landmark-based navigation (Wehner, 2003; Collett et al., 2006; Collett, 2012; Schultheiss et al., 2016).

Landmark based navigation has been widely studied in diurnal ants (Wehner et al., 1996; Fukushi, 2001; Wehner, 2003; Cheng et al., 2009; Collett, 2010; Bühlmann et al., 2011; Wystrach et al., 2011a,b, 2012; Lent et al., 2013; Narendra et al., 2013; Schultheiss et al., 2016; Freas and Cheng, 2017; Freas et al., 2017c), and the current knowledge of landmark use in ants that forage nocturnally is expanding (Reid et al., 2011; Warrant and Dacke, 2011; Freas et al., 2017a,b; Narendra and Ramirez-Esquivel, 2017; Narendra et al., 2017). What these studies have in common is that they explore navigational behavior that occurs chiefly in two dimensions while ants are travelling to goal locations on the ground. Yet foragers of multiple species, most notably those of the *Myrmecia* genus, travel vertically up onto their foraging tree to feed and then must successfully descend to return to the nest (Reid et al., 2011; Narendra et al., 2013; Freas et al., 2017a,b). Nocturnal species of this genus have the added challenge of completing this feat during the evening and morning twilight when visual cues are less salient compared to those used by diurnal species (Reid et al., 2011, 2013; Freas et al., 2017a,b; Narendra et al., 2017).

The study of visually directed behaviour while moving vertically has been little studied outside a few vertebrates (Jeffery et al., 2013; Yartsev and Ulanovsky, 2013). In ant species that forage predominantly on the ground, three-dimensional research has focused primarily on the ability of the path integrator to account for the slope of the ground surface during distance estimation (Wohlgemuth et al., 2001; Wintergerst and Ronacher, 2012). Navigating desert ants appear very adept at integrating terrain slope into their homeward vector, but have not been shown to use landmark cues when foragers are not oriented horizontally. The study of three-dimensional navigation using visual landmark cues is limited to work on the neotropical ant *Cephalotes atratus* L.. This species lives in nests high in the forest canopy, and workers that jump off the trunk direct their fall back to the same tree farther down. These ants have been shown to use landmark-based cues to direct their fall back to the tree trunk, yet appear to orient their bodies horizontally during the fall and may navigate only during this period (Yanoviak et al., 2005; Yanoviak and Dudley, 2006). In the red wood ant, *Formica lugubris*, foragers have been shown to use both chemical and terrestrial cues while ascending and descending trees, yet which terrestrial cues are in use remains unknown (Beugnon and Fourcassié, 1988; Fourcassie and Beugnon, 1988).

Here, we investigate whether foragers of the night-active *Myrmecia midas* actively navigate while foraging vertically on a tree face. *M. midas* foragers rely primarily on landmark cues when navigating to the nest while on the ground (Freas et al., 2017a), and have also been shown to use polarised skylight pattern to compute a homeward vector while on-route (Freas et al., 2017b). However, nothing is known about their behaviour while on a foraging tree. Nests of this species are located in the ground, at the base of a tree trunk. Some individuals forage directly on this ‘nest-tree,’ while other individuals navigate first along the ground before climbing up into a nearby tree’s canopy. First, we examined whether foragers displaced on the vertical tree face position themselves toward the nest direction during their descent to the ground. Next, we tested foragers’ descents when the terrestrial cues and celestial cues were in conflict. Then, we tested a subset of each nest’s foragers that forage on the nest-tree (Freas et al., 2017a). Next, to exclude the use of potential cues beyond the surrounding terrestrial cues, we blocked these terrestrial cues around the nest tree and recorded forager descents without access to the panorama. We also analysed pictures of the visual panorama at different heights and positions on the tree to discover whether nest-oriented views stored while foragers are on the ground contain sufficient information for nest-ward orientation while on the tree. Finally, we describe behaviours foragers exhibit while descending the tree, which appear to be similar to the scanning behaviours previously described on the ground (Wystrach et al., 2014; Zeil et al., 2014).

## Materials and Methods

### Field Site and Study Species

Experiments were conducted from September 2015 to October 2016 on three *M. midas* nests located in forested areas of the Macquarie University campus in Sydney, Australia (33°46′11″ S, 151°06′40″ E; Freas et al., 2017a,b). All three nests were located within a 200 m^2^ area and foragers at each nest foraged on trees within a 15 m radius (typically ≤ 5 m) of the nest entrance. *M. midas* inhabits wooded areas consisting of *Eucalyptus* trees with understories clear of vegetation. All forager collections took place during the evening twilight and all testing occurred during the next morning after sunrise for adequate visibility during testing.

### Foraging Tree Tests

To determine whether foragers travelling on the foraging tree actively navigate to position themselves toward the nest direction during their descent, we collected foragers travelling to a neighbouring foraging tree as they reached the tree base. These individuals were displaced to four sides of the tree face and their homeward paths were observed. This experiment was first conducted on 60 individuals (15 per displacement site) from Nest 1 and then the experiment was repeated on another 40 individuals (10 per displacement site) from Nest 2. During evening twilight, outbound foragers were collected just as they climbed onto their foraging tree located 3 m from the nest entrance at Nest 1 and 4 m from the nest entrance at Nest 2. Foragers were marked with a small amount of paint (Tamiya^TM^, Japan) to prevent retesting. Marked foragers were held overnight in a plastic phial with a small amount of sugar water in a darkened box. The next morning, beginning at 9 am AEST and ceasing at noon, foragers were displaced to one of four sites on the foraging tree face 2 m above ground level. The four displacement sites were designated on the tree face in relation to the nest location (0, 90, 180, and 270°) with 0° being the nest direction and increasing clockwise. Foragers were released from the phial and allowed to climb out of the phial and onto the tree. Once on the vertical tree face, foragers were allowed to return to the nest by climbing down the tree to the ground. As the forager descended the tree, its path was marked at 1 m above ground level, ground level, and 20 cm away from the tree, and directional measurements were recorded at these three points using a smartphone-housed digital compass. Once the forager had travelled 20 cm from the foraging tree it was observed for the remainder of its path to ensure that all individuals returned to the nest entrance.

### Cue Conflict Tests

In our second testing paradigm, we collected 30 foragers at Nest 1 in a similar procedure to the first experiment. Foragers were allowed to leave the nest and travel to their foraging tree located 4 m from the nest entrance. At the base of this foraging tree, these foragers were collected, marked and stored overnight. The next morning, foragers were displaced to the tree located just above nest location (nest tree). It was assumed that these foragers have some previous experience of the panorama at this site due to the proximity to the nest. Foragers were released onto the face of the nest tree, 2 m above ground level, in one of two displacement sites, designated in relation to the nest location (0°, *n* = 15; 180°, *n* = 15) with 0° being the nest direction. This testing regime was conducted on foragers with an acquired homeward vector as ants were captured 4 m from their nest and our displacements put this vector in ∼90° conflict with the terrestrial cues. Identical to previous tests, foragers were released from their phial and allowed to climb onto the nest tree face. Once vertical, foragers were allowed to return to the nest by climbing down the nest tree. As the forager descended the tree, its path was marked at 1 m above ground level and ground level, and directional measurements were recorded at these points. Once ants reached ground level they were observed to ensure all individuals entered the nest.

### Nest Tree Foragers/Landmark Blocking Experiment

The third experiment focused on a subset of ants (*n* = 20) that forage on the tree directly above the nest entrance (Nest 3). These foragers were allowed to leave the nest and travel the short distance to the nest tree (10 cm). Once the forager climbed onto the nest tree at 1.5 m, it was collected in a phial, marked on the gaster to prevent retesting and held overnight with food in an identical procedure to previous tests. The next morning, these foragers were displaced individually onto the nest tree but 180° from the nest direction, 1.5 m from the ground. In this condition, foragers’ full paths on the tree face were recorded by placing small markers just behind the forager as they travelled around the tree face and down to the ground. These markers were placed approximately 10 cm apart along the path and stopped once the individual touched the ground. For each marker, we recorded the height and direction in relation to the nest entrance. Forager paths were calculated at every 10 cm from the release point to the ground and these positions were used for orientation analysis. After testing, foragers were observed as they returned to the nest entrance.

The landmark blocking condition was conducted on a separate group of nest tree foragers at Nest 3 (*n* = 22). Foragers were again allowed to travel the short distance to the nest tree (10 cm). Once the forager climbed onto the nest tree, they were collected, marked and fed, identical to the previous condition. Before testing, (4) 2 m long tent poles were anchored into a 1.5 m × 1.5 m square around the nest tree, ∼75 cm from the tree trunk. A 2 m high thick plastic screen was attached to the pole tops and then anchored to the ground using metal posts. This screen was suspended off the ground by a few centimetres to allow for ants to travel underneath. This set up blocked the surrounding terrestrial cue availability below the 2 m mark on the nest tree, yet did not block the view of the canopy above or any other cues on the nest tree itself. Additionally, nest tree foragers were selected for this condition as the nest entrance was located at the base of the tree (10 cm) and was well within the enclosed square created by the plastic sheet, allowing foragers access to any cues the nest presents. After collection, foragers were displaced on to the tree face opposite the nest site (180°), and 1.5 m off the ground. Foragers’ full paths were recorded using the same methods as in the unblocked condition. After testing, foragers were allowed to search for the nest and upon failure after 3 min. were collected and returned to the correct nest entrance location and allowed to enter the nest.

### Image Analysis: Information Available from the Foraging Tree

For all three nests, we quantified the mismatch in the panoramic scenes between nest-oriented views from the ground at the base of the foraging tree and at different elevations and compass directions on the trees where the ants were tested. To accomplish this, we collected a nest-oriented panoramic image at the base of the foraging tree. We then collected panoramic images at the four cardinal directions on the tree (0, 90, 180, and 270°) at both 1 m and 1.75 m in height. The panoramic image measured 360 pixels width and 117 pixels height (roughly 50 pixels and 67 pixels below and above horizon, respectively) and were down sampled to a resolution of 1 pixel per degree. The images were converted to grayscale by keeping the blue colour channel only. This diminishes differences between clouds and blue sky but maintains high contrasts between terrestrial objects and the sky. Rotational image difference functions (rotIDFs) were calculated by using the sum of the absolute difference in pixel intensity between the reference and test images, for all possible rotations of the test images (in one-degree steps) using custom written scripts in MATLAB (for further details, see Zeil et al., 2003, 2014; Stürzl and Zeil, 2007).

### Scanning Behaviour

In order to describe the scan-like behaviour on the tree face, individual foragers were recorded both while on the tree face after displacement and on a vertically oriented board. Forager scans were recorded using a free held camera (PowerShot G12, Canon^TM^). Foragers were recorded after local off-route displacement on their foraging tree.

### Statistical Procedure

Data from all experiments were analysed with circular statistics (Batschelet, 1981; Zar, 1998) using the statistics package Oriana Version 4 (Kovach Computing Services^TM^). Rayleigh’s Tests were conducted on foragers’ positions on the tree face, testing if data met the conditions of a uniform distribution (*p* > 0.05). If data were not uniform, we tested whether positioning on the tree face was significantly clustered around the nest direction using *V*-tests, with alpha set at *p* = 0.05. We also examined if the predicted direction (0°) fit within the 95% confidence interval of the foragers’ positions during descent to further test positioning toward the nest (Watson Test). When an ant abandoned its descent to travel back up the tree (see blocking condition), only the positions of the individual’s final descent were used for analysis.

## Results

Individuals placed on the tree face at the displacement sites initially paused for a short period. After this pause, foragers typically moved a short distance (usually up the tree 10–30 cm) away from the displacement point and then paused again and performed what we classify as scanning behaviours on the tree face (described below). Following this scanning behaviour, the forager moved along the tree face descending to the ground. During their descent, foragers typically performed at least one more scan-like behaviour before reaching the ground.

### Foraging Tree Tests

**FIGURE 1:**
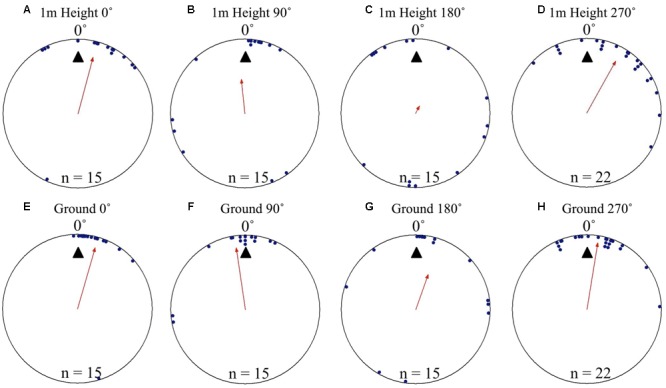
Circular distributions of individual *Myrmecia midas* foragers’ positions on the tree face during displacement experiments on their foraging tree. Figures show the raw data of forager positions at two heights after displacements to one of four sides of the tree at a 2 m height at Nest 1. The nest direction for each figure is at 0°, labelled by a black triangle. The arrow denotes the direction and length of the mean vector. Foragers were collected at the base of their foraging tree, held overnight and then released vertically on the tree face at one of four sites (0, 90, 180, and 270°). **(A)** The position of individual foragers released at the 0° location at 1 m in height. **(B)** Forager positions of individuals released at the 90° location at 1 m in height. **(C)** Forager position of individuals released at the 180° location at 1 m in height. **(D)** Forager positions of individuals released at the 270° location at 1 m in height. **(E)** The position of individual foragers released at the 0° location as they reach the ground. **(F)** Forager positions of individuals released at the 90° location as they reach the ground. **(G)** Forager position of individuals released at the 180° location as they reach the ground. **(H)** Forager positions of individuals released at the 270° location as they reach the ground.

At both the 1m height and as they reached the ground at 0 m, Nest 1 foragers’ positions on the tree face in the 0, 90, and 270° displacement conditions were non-uniform and significantly clustered to the nest’s direction at 0°. Additionally, in these three conditions at both heights (1 and 0 m), the nest direction fell within the 95% confidence interval of the forager’s positions (**Table 1** and **Figures 1A,B,D–F,H**). In the 180° condition, foragers’ positions when crossing the 1 m height were uniform and not directed to the nest direction at 0° (**Table 1** and **Figure 1C**). Yet as foragers in the 180° condition reached the ground, their positions on the tree were significantly non-uniform and clustered to the nest’s direction at 0°. The nest direction also fell within the 95% confidence interval of the foragers’ positions at 0 m (**Table 1** and **Figure 1G**). After reaching 20 cm from the tree base, forager paths in all four conditions at Nest 1 were grouped toward the nest entrance (**Table 1**) and all individuals immediately travelled the 3 m back to and entered the nest.

**Table 1:** Statistical results for all on tree displacement conditions.

Conditions	Mean vector μ (°)	95% Confidence Interval		Rayleigh test		*V*-test: direction 0°	
		Minus (°)	Plus (°)	*Z*	*p*	*V*	*p*
**Foraging tree**
**Nest 1**
1 m height 0**°**	14.811	352.279	37.342	9.101	<0.0001	0.753	<0.0001
1 m height 90**°**	354.022	309.132	38.912	3.179	0.039	0.458	0.005
1 m height 180**°**	27.744	–	–	0.175	0.844	0.096	0.302
1 m height 270**°**	29.118	12.786	45.45	13.672	<0.0001	0.689	<0.0001
Ground 0**°**	15.914	357.611	34.218	10.838	<0.0001	0.817	<0.0001
Ground 90**°**	351.419	332.242	10.595	10.479	<0.0001	0.826	<0.0001
Ground 180**°**	19.445	338.546	60.344	3.667	0.023	0.466	0.005
Ground 270**°**	9.096	358.143	20.05	17.834	<0.0001	0.889	<0.0001
**Nest 2**						
1 m height 0**°**	339.238	306.629	11.848	5.391	0.002	0.687	0.0006
1 m height 90**°**	356.192	326.402	25.981	5.951	0.001	0.77	<0.0001
1 m height 180**°**	352.377	323.045	21.71	6.045	0.0009	0.771	<0.0001
1 m height 270**°**	334.418	301.121	7.715	5.261	0.003	0.654	0.001
Ground 0**°**	355.578	328.26	22.896	6.466	0.0004	0.802	<0.0001
Ground 90**°**	1.842	333.859	29.825	6.326	0.0005	0.795	<0.0001
Ground 180**°**	353.449	345.539	1.36	9.648	<0.0001	0.976	<0.0001
Ground 270**°**	346.333	327.786	4.88	8.209	<0.0001	0.88	<0.0001
**Non-foraging tree**
**Nest 3**
1 m height 0**°**	12.233	343.921	40.546	6.233	0.001	0.63	0.0002
1 m height 180**°**	355.73	331.507	19.952	8.435	<0.0001	0.748	<0.0001
Ground 0**°**	358.238	350.913	5.563	14.245	<0.0001	0.974	<0.0001
Ground 180**°**	0.914	337.407	24.422	8.713	<0.0001	0.762	<0.0001
**Blocking condition**
**Nest 3**
Unblocked 1.4 m height 180**°**	153.717	235.053	72.381	0.081	0.924	–0.057	0.64
Unblocked 1 m height 180**°**	351.434	314.573	28.295	4.14	0.014	0.45	0.002
Unblocked ground 180**°**	1.516	351.661	11.372	17.141	<0.0001	0.925	<0.0001
Blocked 1.4 m height 180**°**	112.55	5.152	219.947	0.539	0.589	–0.063	0.653
Blocked 1 m height 180**°**	148.008	336.265	319.752	0.213	0.812	–0.087	0.708
Blocked ground 180**°**	213.213	133.851	292.575	0.977	0.381	–0.185	0.878

**FIGURE 2:**
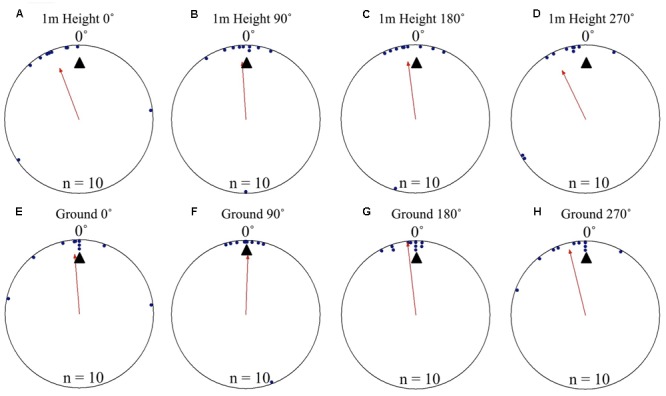
Circular distributions of individual *M. midas* foragers’ positions on the tree face during displacement experiments on their foraging tree. Figures show the raw data of forager positions at two heights after displacements to one of four sides of the tree at a 2 m height at Nest 2. The nest direction for each figure is at 0°, labelled by a black triangle. The arrow denotes the direction and length of the mean vector. Foragers were collected at the base of their foraging tree, held overnight and then released vertically on the tree face at one of four sites (0, 90, 180, and 270°). **(A)** The position of individual foragers released at the 0° location at 1 m in height. **(B)** Forager positions at Nest 2 of individuals released at the 90° location at 1 m in height. **(C)** Forager position at Nest 1 of individuals released at the 180° location at 1 m in height. **(D)** Forager positions at Nest 1 of individuals released at the 270° location at 1 m in height. **(E)** The position of individual foragers released at the 0° location as they reach the ground. **(F)** Forager positions of individuals released at the 90° location as they reach the ground. **(G)** The position of individual foragers released at the 180° location as they reach the ground. **(H)** Forager positions of individuals released at the 270° location as they reach the ground.

At Nest 2, foragers’ positions on the tree face in all displacement conditions (0, 90, 180, and 270°) were significantly non-uniform and significantly clustered to the nest’s direction at 0° as they crossed to the 1 m height marker. Additionally, the nest direction fell within the 95% confidence interval of the foragers’ positions at 1 m high in all conditions (**Table 1** and **Figures 2A–D**). Nest-ward positioning continued as foragers reached the ground, with all conditions showing significant non-uniformity and significant cluster toward the nest direction. Additionally, the nest fell within the 95% confidence interval of the foragers’ positions (**Table 1** and **Figures 2E–H**). At Nest 2, once foragers had reached 20 cm from the tree, all individuals were oriented to the nest direction at 0° (**Table 1**), travelled in a straight path to the nest entrance and entered.

At the ground, foragers typically did not stop to scan again but continued on in their current direction. In all conditions foragers immediately returned to the nest entrance and entered the nest.

### Cue Conflict Tests

**FIGURE 3:**
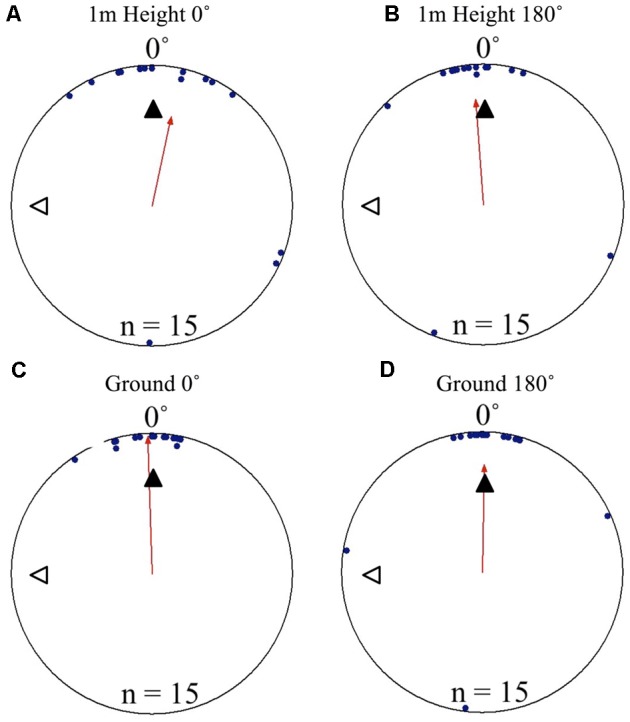
Circular distributions of individual *M. midas* foragers’ positions on the tree face during displacement experiments with cue conflicts. Figures show the raw data of forager positions at two heights after displacements to one of two sides of the tree at a 1.5 m height at Nest 3. The nest direction for each figure is at 0°, labelled by a black triangle. The foragers’ accumulated vector was at 270°, labelled by a white triangle. The arrow denotes the direction and length of the mean vector. Foragers were collected at the base of their foraging tree, held overnight and then released vertically on the tree face of the nest tree at one of two sites (0 and 180°). **(A)** The position of individual foragers released at the 0° location at 1 m in height. **(B)** Forager position at Nest 1 of individuals released at the 180° location at 1 m in height. **(C)** The position of individual foragers released at the 0° location as they reach the ground. **(D)** The position of individual foragers released at the 180° location as they reach the ground.

To test if foragers position themselves toward either the terrestrial or celestial cues during their decent, we displaced foragers off their foraging route in order to put these cue sets in 90° conflict. Individuals foraging away from the nest and displaced on the nest tree showed significant nest directed positioning on the tree face at 1 m above ground level. Positions on the tree in both the 0 and 180° displacement conditions were significantly non-uniform and significantly grouped to the nest direction at 0°. This pattern continued as the foragers reached the ground, with foragers’ positions being significantly directed to the nest location and non-uniform. In both conditions and at both the 1 m height and at ground level (0 m), the nest direction fell within the 95% confidence interval of foragers’ positions on the tree (**Table 1** and **Figures 3A–D**). Foragers in both the 0 and 180° conditions showed no evidence of using their celestial based vector while positioning themselves on the tree (at 270°). After descending the tree, all foragers found and entered the nest (15 cm from the tree). At the ground, foragers continued on in their current direction. In all conditions foragers immediately returned to the nest entrance and entered the nest.

### Nest Tree Foragers/Landmark Blocking Experiment

**FIGURE 4:**
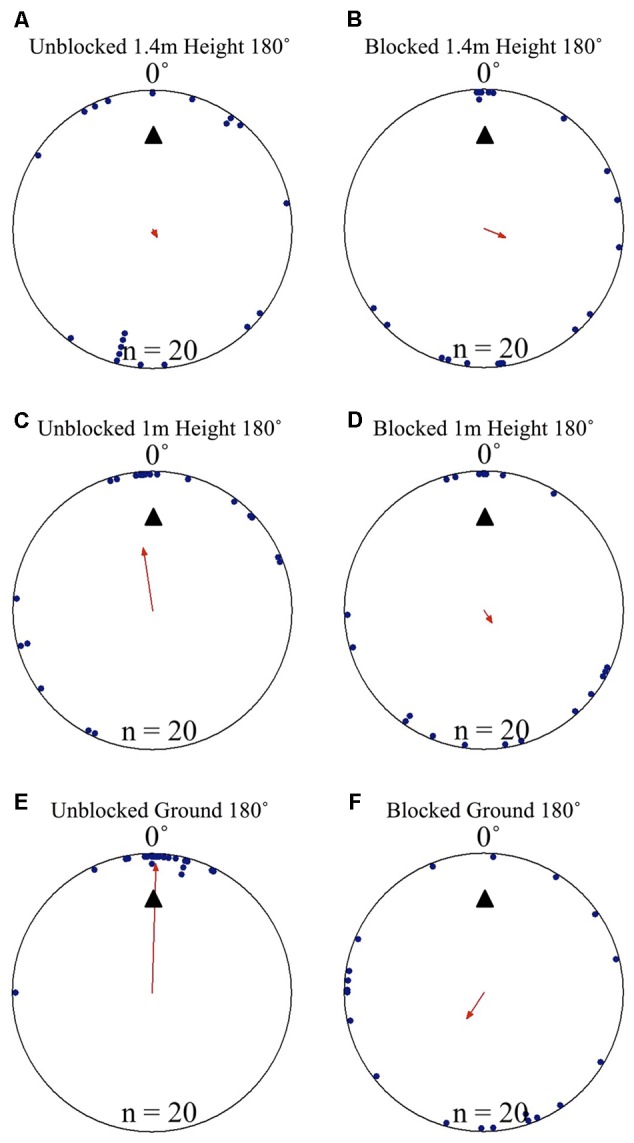
Circular distributions of individual *M. midas* nest tree foragers’ positions on the tree face during the landmark blocking experiments on the nest tree. Figures show the raw data of forager positions at three heights after displacements to one of two sides of the tree at a 1.5 m height at Nest 3. The nest direction for each figure is at 0°. The arrow denotes the direction and length of the mean vector. Foragers were collected at the base of the nest tree, held overnight and then released vertically on the tree face of the nest tree opposite the nest entrance (180°) with the surrounding landmark panorama either unblocked or blocked. **(A)** The position on the tree face of individual foragers released at the 180° location as they begin their descent at 1.4 m in height with the surrounding landmarks unblocked. **(B)** The position on the tree face of individual foragers released at the 180° location as they begin their descent at 1.4 m in height with the surrounding landmarks blocked. **(C)** The position on the tree face of individual foragers released at the 180° location at 1 m in height with the surrounding landmarks unblocked. **(D)** The position on the tree face of individual foragers released at the 180° location at 1 m in height with the surrounding landmarks blocked. **(E)** The position on the tree face of individual foragers released at the 180° location as the forager reaches the ground with the surrounding landmarks unblocked. **(F)** The position on the tree face of individual foragers released at the 180° location as the forager reaches the ground with the surrounding landmarks blocked.

**FIGURE 5:**
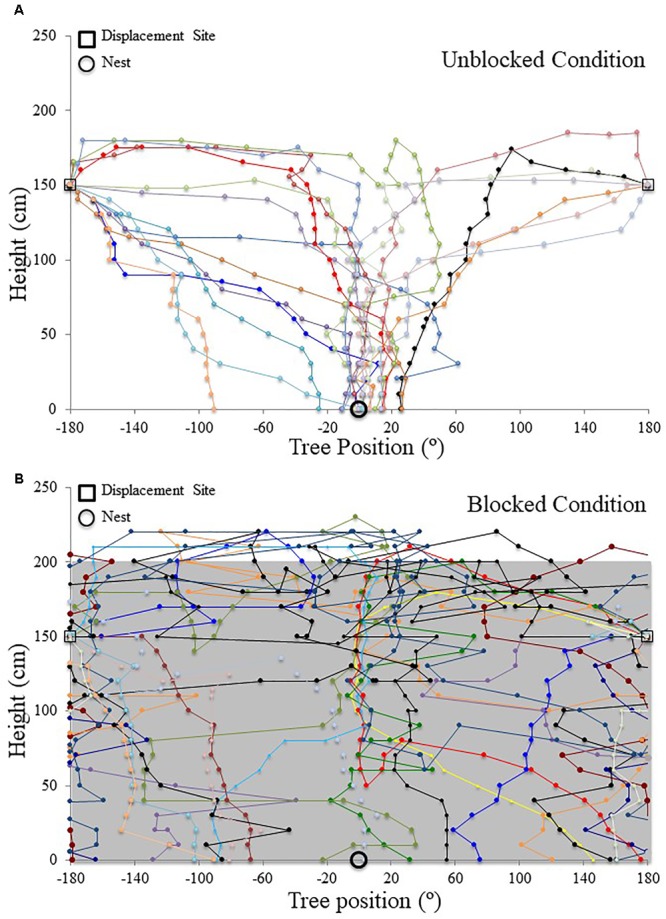
Individual *M. midas* nest tree foragers’ paths descending the tree face in the landmark blocking experiment. Circular positions on the tree face have been unwrapped to show individuals’ paths from the 180° off-route, 1.5 m high displacement site (open square) to the ground. The plots are cylindrical, with +180° and –180° being the same position on the side of the tree opposite the nest. The open circle at ground level (0 cm) denotes the nest entrance direction. **(A)** Forager paths in the unblocked condition with the surrounding landmarks visible. **(B)** Forager paths in the blocked condition with all surrounding landmarks below 2 m blocked using a plastic screen. The grey area in the background signifies the blocking screen surrounding the tree from 0 to 2 m.

Nest tree foragers displaced to the opposite side of the tree (180°) from the nest tree at 1.5 m with access to the surrounding terrestrial cues behaved similarly to foragers that travel away from the nest to forage on a different tree. Foragers initially paused at the release point, and then moved a small distance, where they performed scan-like behaviours. These continued intermittently during the forager’s decent. At the 1.4 m height, after a 10 cm decent, foragers showed uniform positioning around the tree and were not oriented to the nest site (**Table 1** and **Figures 4A, 5A**). This uniform distribution continued at the 1.3 m, and 1.2 m heights (Rayleigh test, *P* > 0.05; *V*-test, *P* > 0.05). At 1.1 m, forager positions were still uniform (Rayleigh test, *Z* = 1.754, *P* = 0.174) but were significantly clustered to the nest direction, and the nest location was within the 95% confidence interval of forager positions (*V*-test, *V* = 0.295, *P* = 0.031). At the 1 m height, forager positions on the tree face became significantly non-uniform and significantly grouped around the nest direction at 0° (**Table 1** and **Figures 4C, 5A**). This non-uniform and clustered pattern persisted at all 10 cm height measurements from 1 m to ground level with foragers significantly positioned on the nest side of the tree (1 m – 0 m; Rayleigh test, *P* < 0.001; *V*-test, *P* < 0.001; **Table 1** and **Figures 4E, 5A**). At all heights between the 1 m and ground level measurements, the nest direction fell within the 95% confidence interval of foragers’ positions on the tree. Once foragers had completed their descent, all individuals found and entered the nest (10 cm from the tree).

When the surrounding terrestrial cues were blocked, nest-tree foragers displaced to the opposite side of the tree (180°) behaved differently from previous conditions. Foragers typically scanned once near the displacement point. After this, half of the foragers tested (*n* = 10) travelled up the trunk above the 2 m-blocked height before beginning to perform more scans. As a whole (*n* = 20), foragers did not orient to the correct nest direction at any height 1.4–0 m during their descent (1.4, 1, and 0 m; **Table 1** and **Figures 4B,D,F, 5B**). At all heights, forager positions on the tree met conditions of a uniform distribution (1.4 – 0 m, Rayleigh test, *P* > 0.05) and were not significantly oriented in the direction of their home vector at 0° (1.4–0 m, *V*-test, *P* > 0.05). As foragers reached the ground, they did not travel to the nest entrance located within the landmark-blocking arena but instead performed looping paths, some even returning back up the tree. After 3 min, two individuals found the nest entrance and the rest were collected and moved to the nest entrance where they willingly entered.

Focusing only on those foragers that responded to the blocked panorama by ascending the tree to 2 m or higher (**Figure 5B**), when foragers first descended from 2 m or higher, they were positioned toward the nest site at 190 cm (*V*-test, *V* = 0.745, *P* < 0.001). This nest-ward positioning continued at all heights through 1.4 m height (*V*-test, *V* = 0.578, *P* = 0.004) until the 1.1 m height where forager positions were no longer non-uniform (Rayleigh test, *Z* = 0.504, *P* = 0.616) and no longer clustered to the nest side of the tree (*V*-test, *V* = 0.203, *P* = 0.186). These foragers’ positions were uniform and not clustered toward the nest at any height between 1 m (Rayleigh test, *Z* = 0.559, *P* = 0.583; *V*-test, *V* = 0.132, *P* = 0.282) and 0 m (Rayleigh test, *Z* = 0.974, *P* = 0.387; *V*-test, *V* = -0.177, *P* = 0.782). Foragers that did not ascend above the blocking screen (*n* = 10) were not positioned toward the nest at any height (*V*-test, 1.4 m, *V* = -2.827, *P* = 0.988; 1 m, *V* = -1.474, *P* = 0.929; 0 m, *V* = -0.862, *P* = 0.802).

### Panoramic Image Analysis: Information Available from the Foraging Tree

**FIGURE 6:**
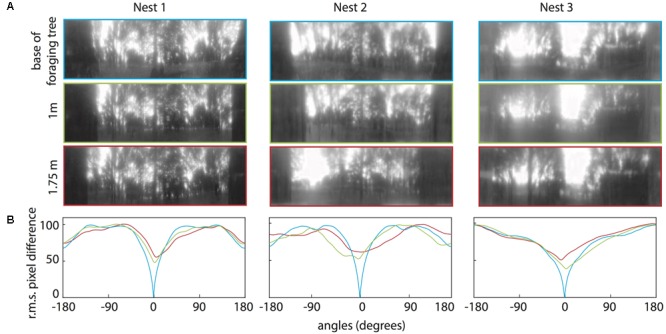
Quantifying the change in the panorama at different elevations on the foraging tree at the three nests. **(A)** Panoramic images at the base of the foraging tree (blue), 1 m in height (green), and 1.75 m in height (red). Images were downscaled to 1 pixel per 1° to resemble the ant’s visual acuity, filtered through only the blue colour channel and oriented with the nest centred. **(B)** The rotIDF compares the root mean square pixel difference between the panorama at the base of the foraging tree with itself (blue), the 1 m (green), and the 1.75 m (red) panoramas. The nest direction in all comparisons is centred at 0°.

**FIGURE 7:**
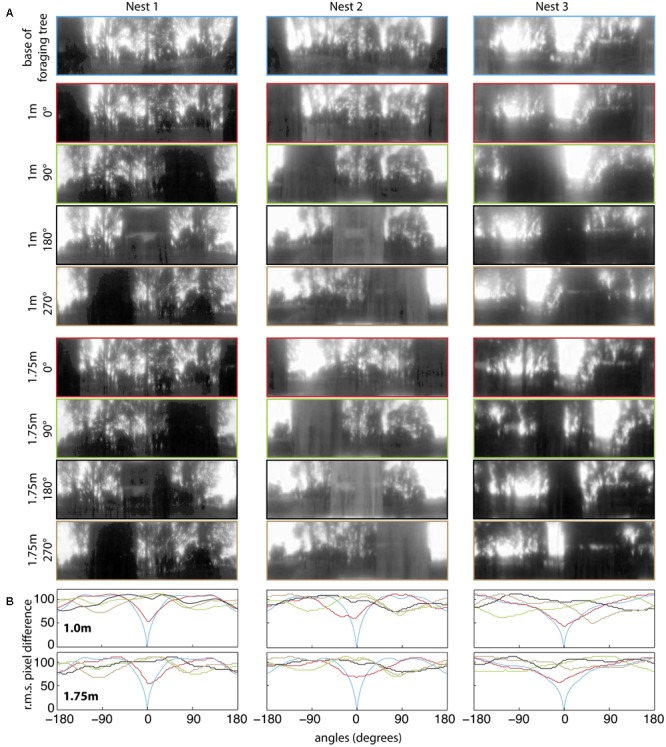
Quantifying panorama changes at the four displacement directions and at two elevations on the foraging tree at the three nests. **(A)** Panoramic images at the base of the foraging tree (blue), 1 m in height at 0° (red), 90° (green), 180° (black), 270° (orange) and 1.75 m in height at 0° (red), 90° (green), 180° (black), 270° (orange). Nest orientation is at the centre of each image and images were downscaled to 1 pixel per 1° to resemble the ant’s visual acuity, filtered through only the blue colour channel and oriented with the nest centred. **(B)** The rotIDF compares the root mean square pixel difference between the panorama at the base of the foraging tree with itself, and the foraging tree at both 1 and 1.75 m at each direction. The nest direction in all comparisons is centred at 0°.

For all three nests, when comparing the nest-oriented panoramic views from the base of the tree to nest-oriented panoramic views at 1 m and 1.75 on the tree, we found that at both heights on the tree, the rotIDFs showed a distinct valley of minimum of mismatch (i.e., best matching direction) that was directed toward the nest [**Figures 6A,B** (green and red curves)]. This shows that directional information can be recovered up to 1.75 m (at least) from a visual memory acquired at the base of the foraging tree. We then analysed whether animals can recover nest oriented views from different compass directions around the tree (0° = nest). At both 1 and 1.75 m on the tree, the views available at the other directions, 90° (green), 180° (black), and 270° (brown), do not generate a clear minima when compared with a view at the base of the tree (**Figures 7A,B**).

### Scanning Behaviour

While ants were on the tree face, foragers exhibited several kinds of scanning behaviours, the common characteristic of which was a shift of the body and head to bring the head’s orientation at or near the horizontal plane. With the head at or close to horizontal, individuals then slowly rotated their head horizontally across the field.

**FIGURE 8:**
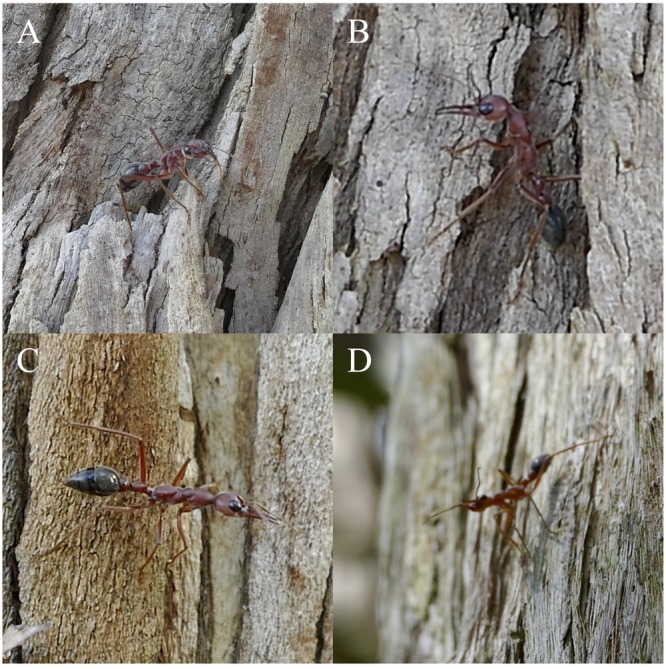
The four described vertical scanning behaviours. All images were taken as foragers were descending their foraging tree after displacement. **(A)** The horizontal scan. **(B)** The downward pitch scan. **(C)** The head roll scan. **(D)** The push up or upward pitch scan.

The first kind of scan-like behaviour exhibited by these foragers was to use a piece of the tree’s structure, such as a jutting piece of bark, a knot, or burl, creating a horizontal space at the top at which individuals can orient their entire body horizontally and then slowly shift their head across the horizontal plane (**Figure 8A**). This behaviour was environment-dependent and could occur at any point during the foragers’ descent.

The second kind of scan-like behaviour, dubbed downward pitch scans, occurred as the individual reached the top of a bark strip or other structure and was oriented upward. Individuals lowered the pitch of their head while the body remained vertical, allowing individuals to bring the head close to the horizontal plane (**Figure 8B**). This behaviour was also environment-dependent but typically occurred during the initial portion of the foragers’ route when some foragers travelled upward from the displacement site.

The third kind of scan-like behaviour, termed head roll scans, occurred as foragers were travelling horizontally across the vertical tree face. Foragers altered their head position by rolling the head toward the tree face, bringing the tree side of their head down and positioning their head close to the horizontal plane. From here, individuals slowly moved their head across the horizontal plane to scan (**Figure 8C**). This behaviour typically occurred when foragers were not yet on the nest side of the tree.

The final kind of scan-like behaviour, labelled the push up or upward pitch scan, was observed on the vertical tree face with the individual oriented down with the head positioned below the body. The individual extended its front legs, pushing its body and head away from the tree face. The individual’s head pitched upward, reaching at or near the horizontal plane. In this position, the individual would slowly move its head across the field (**Figure 8D**). The upward pitch scan was usually observed as foragers reached the side of the tree facing the nest. These behaviours would continue throughout the forager’s descent when on their descending route.

## Discussion

In the current study, we show that *M. midas* foragers successfully orient to the nest side of their foraging tree during their descent. Correct nest directed positioning appears to occur well before foragers reach the ground, with foragers’ positions grouped toward the nest direction at the 1-m height and at ground level. This ability appears to extend beyond the forager’s current foraging tree as individuals displaced from their foraging tree to the nest tree also successfully positioned themselves toward the nest direction both at 1-m height and at ground level. Even nest-tree foragers, which show evidence of reduced navigational knowledge on the ground (Freas et al., 2017a), are able to successfully orient while on their foraging tree above the nest entrance. Visual terrestrial cues appear to be critical to this navigational ability, as when the surrounding terrestrial cues were blocked, foragers were unable to successfully orient toward the nest entrance. Analysis of the panorama at different foraging heights suggests that ants can obtain nest orientation information at both 1 and 1.75 m above the ground, provided they are on the nest-facing tree face (0°). Finally, use of the surrounding terrestrial cues fits with behaviour on the tree as foragers appear to actively scan while on the tree, bringing their head orientation to or near the horizontal plane and then slowly rotating it across the field.

When *M. midas* foragers are displaced in a local environment on the ground, they are able to successfully use the surrounding landmark cues to orient toward the nest (Freas et al., 2017a). Our results suggest this ability extends to elevation-based displacements. The ability to orient to familiar landmarks after vertical displacement has been previously shown in the desert ant *M. bagoti* (Schwarz et al., 2014), a species that forages on the ground almost exclusively (Schultheiss and Nooten, 2013). It is currently unknown if foragers include travelling vertically up the nest tree in their learning walks or if on their first trip onto the foraging tree they perform a vertical form of turn back behaviour as is observed with ants on the ground (Nicholson et al., 1999; Graham and Collett, 2006; Müller and Wehner, 2010; Fleischmann et al., 2016, 2017) and has also been reported in bees (Lehrer, 1991, 1993).

Similar nest-ward positioning was present when foragers were displaced off their foraging route to the nest tree. Ant species inhabiting complex, landmark-rich environments typically rely heavily on terrestrial cues for navigation, with landmarks tending to suppress any accumulated vector information (Wehner et al., 1996; Narendra, 2007; Narendra et al., 2013; Mangan and Webb, 2012). Yet in situations where the celestial based vector and terrestrial cues conflict, some species exhibit directional compromise behaviour (Narendra, 2007; Collett, 2010; Legge et al., 2014; Wystrach et al., 2015; Wehner et al., 2016). This compromise between cues sets has not been observed in *M. midas* while navigating on the ground, as terrestrial cues largely dominate in a local area (Freas et al., 2017a). Yet *M. midas* foragers have shown evidence of vector cue use and celestial/terrestrial directional cue compromise while on their foraging route during both the outbound and inbound journeys (Freas et al., 2017b). In the current study, foragers showed similar behaviour with no evidence of using their naturally accumulated celestial based vector for positioning and their behaviours were consistent with navigation through terrestrial cues. It is worth noting that the accumulated vector lengths in this test are relatively short (4 m), but this distance is representative of the typical vector length by observed individuals at our field site (Freas et al., 2017a) and foragers have been shown to use celestial cues at these distances (Freas et al., 2017b).

The final unblocked condition tested foragers that travel straight up the nest tree to forage. These foragers have been previously shown to be unable to successfully orient when displaced locally on the ground (Freas et al., 2017a). It is believed that these foragers are naturally restricted horizontally to the nest site and either do not actively navigate during foraging or have reduced navigational abilities similar to *C. bicolor* digger ants, which do not forage (Wehner and Menzel, 1969; Freas et al., 2017a). The results of our unblocked condition suggest these foragers do actively navigate while foraging in the nest tree as these individuals successfully orient to the nest side of their foraging tree after displacement and this positioning occurs well before they reach the ground.

Our landmark blocking condition also tested nest-tree foragers, allowing us to keep the nest entrance and any directional cues it provides within the blocking arena and accessible to the foragers. Foragers’ inability to position themselves toward the nest direction in this setup corresponds with landmark blocking experiments on the ground where foragers cannot orient to the nest when the surrounding panorama is blocked (Freas et al., 2017a). These results would also appear to exclude any scent-based cue, or local visual cues on the tree surface that could be used on their own for directional information. Our results also suggest that this species cannot use the unblocked canopy of the tree alone for directional information, at least during the final 2 m of their decent.

The use of the surrounding panorama for direction information is also supported by forager behaviour in the blocking condition before descending the tree. Foragers that immediately descended the tree (*n* = 10) were not positioned toward the nest at any height as expected if foragers used the surrounding terrestrial cues to orient. Foragers (*n* = 10) that responded to the blocking screen by first ascending above 2 m were positioned correctly but below 1 m correct positioning ceased (1-0 m). These findings suggest that the distant terrestrial cues are critical not only for a forager’s initial positioning but are also involved in route maintenance during a forager’s descent. It is possible that foragers must scan the surrounding visual panorama during their descent in order to maintain positioning on the tree. This would explain the scanning behaviour observed throughout forager descents in all conditions.

Our analysis of panoramic pictures revealed that sufficient visual information is available in the scene for the ants to orient on these trees. Image comparisons revealed variability across trees and locations, but overall, the information necessary to retrieve the nest direction using a terrestrial visual compass strategy (Wystrach et al., 2011b; Baddeley et al., 2012) is available. As noted earlier (Zeil et al., 2003; Schwarz et al., 2014), changes in height have little impact on the information available in these panoramic views. This stable nest-ward minimum in panorama information may also be used by bees and wasps as they ascend in height during their learning flights (Zeil, 1993a,b; Stürzl et al., 2016; Murray and Zeil, 2017). In the case of our ants, it is worth noting that using memories from the correct side of the tree is useful primarily when the ant is currently located on that side of the tree, as this position was where the best matches were obtained. It appears that rotIDF is not very powerful at predicting the nest direction when the ant is located on an unfamiliar side of a tree (90, 180, or 270°), but has more predictive power when the ant is located on the familiar side (0°). Even though there was no detectable minima at the 90, 180, or 270° positions on the tree (**Figure 7B**), ants were able to successfully guide themselves back toward their familiar corridor on the tree and then toward the nest. This reflects what is observed on the ground. Assuming that ants learn the scene when located on their habitual side of the tree, this would provide a gradient of familiarity that could be used to reach and stick stay on the nest side of the tree. Whether foragers use this gradient of familiarity (Zeil et al., 2003), the visual compass (Wystrach et al., 2011b; Baddeley et al., 2012) or other visual strategies (Wystrach et al., 2012; Horst and Möller, 2017), remains to be tested.

Scanning behaviour characterised by the rotation of the individual’s head and body in place (Wystrach et al., 2014; Zeil et al., 2014) can be useful to exploit the familiarity of the surrounding visual scene. Ants perform more scans when their familiar surroundings have been altered or when the direction provided by terrestrial cues conflicts with celestial cues (Wystrach et al., 2014). In the current study, we show that this behaviour may extend beyond ground level, as individuals travelling vertically appear to actively scan while on their foraging tree. This potential behaviour, which is closely associated with the use of learnt visual cues, along with the results of the blocking condition and the panorama analysis, further indicate that the use of learnt visual cues is likely in use during forager descents. It has recently been shown that while on their foraging route members of *M. pyriformis*, another nocturnal *Myrmecia* species that relies heavily on the visual scene (Reid et al., 2011), attempt to stabilise their head horizontally while travelling en route on an uneven surface, as view similarity drops markedly as the view is rotated (Raderschall et al., 2016). This species has also been shown to perform extensive scanning behaviours during learning walks around the nest indicating scan behaviours are part of the nocturnal ant’s navigational repertoire (Narendra and Ramirez-Esquivel, 2017). Similar behaviours seem to apply to navigation on the tree in *M. midas* where foragers appear to attempt through multiple scanning behaviours to position their heads horizontally during scanning. These scans may serve a similar function as scans displayed on the ground (Wystrach et al., 2014; Narendra and Ramirez-Esquivel, 2017), and thus suggest that similar visual memories and strategies may be used when foraging both on ground and on trees. A future study on the foragers’ ability to effectively scan while navigating along a vertical plane is warranted.

It is also important to note that the described behaviour of raising the head while vertical may also potentially involve the use of celestial cues, such as the sun’s position, when they are available. Work on honeybee dancing in the Asian species *Apis florea*, a behaviour strongly tied to the position of the sun, has shown that when dancers are on a steep slope, these individuals rotate their head position to compensate for this slope. This compensation allows them to keep their visual field stable with the horizon while dancing (Dyer, 1985, 2002). This behaviour appears similar to what we observe in the current study, albeit without the horizontal movement of the head, which we have deemed scanning behaviour. It remains possible that foragers could also be using celestial cues as well as terrestrial cues while on the tree. *M. midas* foragers typically only forage in trees within 5 m of the nest and have shown no evidence of orienting to vectors of this length. In the rare case that foragers travel farther from the nest (14 m), we have only observed weak evidence of orientation to a vector (Freas et al., 2017a). As such, it may be possible that the observed scanning behaviour on the tree surface also allows foragers access to celestial cues.

Finally, the extent of these vertical navigational abilities is currently unknown, as well as at what height these individuals naturally show nest ward positioning during their descent. Observations of returning foragers in the predawn twilight suggest that foragers are oriented to the nest at heights over 3 m, yet an analysis of this behaviour may prove difficult. *M. midas* nests at the field site are located in small stands of trees, interspersed with large tracks of grass. This habitat leads to large differences in skyline height surrounding the nest. These large skyline changes may not change drastically with changes in height of the viewer. Further studies into how the terrestrial cues change over larger changes in elevation are warranted.

## Conclusion

The experiments in the current study show that *M. midas* actively and critically use the surrounding visual scene to orient and descend along the correct side of the tree. Image analysis of the visual scene on the tree shows that the scene provides sufficient information for these individuals to orient successfully using stored views. These foragers may extract this visual information during on-tree scanning behaviours where individuals scan their surroundings in the horizontal plane. Together, these findings suggest that visual navigational strategies and memory use may be similar between foragers navigating on the ground and on the tree.

## Author Contributions

Experiments and analyses were designed by CF, AW, AN, and KC. CF collected all data. CF, AN, and AW analysed the data. CF, AW, AN, and KC drafted and revised the manuscript.

## Conflict of Interest Statement

The authors declare that the research was conducted in the absence of any commercial or financial relationships that could be construed as a potential conflict of interest.
